# How Did Italian Adolescents with Disability and Parents Deal with the COVID-19 Emergency?

**DOI:** 10.3390/ijerph18041687

**Published:** 2021-02-10

**Authors:** Silvia Faccioli, Francesco Lombardi, Pierantonio Bellini, Stefania Costi, Silvia Sassi, Maria Cristina Pesci

**Affiliations:** 1 PhD Program in Clinical and Experimental Medicine—Department of Biomedical, Metabolic and Neural Sciences, University of Modena and Reggio Emilia, 41125 Modena, Italy; silvia.faccioli@ausl.re.it; 2 Children Rehabilitation Unit of S. M. Nuova Hospital, Azienda Unità Sanitaria Locale—IRCCS di Reggio Emilia, 42123 Reggio Emilia, Italy; silvia.sassi@ausl.re.it (S.S.); mariacristina.pesci@ausl.re.it (M.C.P.); 3 Neurorehabilitation Unit of S. Sebastiano Hospital, Azienda Unità Sanitaria Locale—IRCCS di Reggio Emilia, 42015 Reggio Emilia, Italy; francesco.lombardi@ausl.re.it; 4 Unit of Dentistry and Oral-Maxillo-Facial Surgery, Department of Surgery, Medicine, Dentistry and Morphological Sciences with Transplant Surgery, Oncology and Regenerative Medicine Relevance—University of Modena and Reggio Emilia, 41124 Modena, Italy; pierantonio.bellini@unimore.it; 5 Scientific Directorate, Azienda Unità Sanitaria Locale—IRCCS di Reggio Emilia, 42123 Reggio Emilia, Italy

**Keywords:** COVID-19, disability, rehabilitation, anxiety, survey, lockdown

## Abstract

The COVID-19 emergency has imposed distanced education and has interrupted most rehabilitation services. Adolescents with disabilities have been isolated, and the burden on their families has been exacerbated. A cross-sectional survey was administered to adolescents with disability and to parents of disabled children to describe their experience during lockdown and their concerns or expectations about rehabilitation. A sample of 53 adolescents and 239 parents completed the survey. Adolescents were ages 13–18 years old (45.3% female). Most parents were between 35 and 55 years old (84.9% female). While 53.6% of the parents reported no positive effects of the lockdown, 92.5% of the adolescents expressed favorable consequences. The increased time spent with family members was judged positively by 27.2% of parents and by 64.2% of adolescents. Concern for their child’s disability was expressed by 47.3% of parents, while 73.6% of adolescents expressed concerns regarding the ban on meeting friends. In both groups, anxiety symptoms were correlated with the fear of contracting COVID-19 and with financial problems. Parents would have liked even more remote support from school and healthcare professionals, which was available for most participants. Thus, socioeconomic support, assistive technology and telerehabilitation strategies might help families with disabilities during a lockdown.

## 1. Introduction

The COVID-19 global pandemic has dominated the international scene since March 2020. This acute respiratory syndrome, often asymptomatic but potentially lethal, is caused by the spread of the SARS-CoV-2 virus, first seen in China [1]. According to the World Health Organization, there were 162,488 confirmed cases in Italy on 15 April 2020, with a total of 21,069 deaths. At that time, Italy was the third leading country in terms of positive cases in the world (after the U.S.A. and Spain) and the second leading country in Europe (WHO Situation Report-86). During the first wave of the pandemic, northern Italy saw a rapid, massive diffusion of COVID-19, which monopolized healthcare resources, resulting in a dramatic reduction in those healthcare services that could be deferred.

After the lockdown imposed by the Italian Prime Minister [2], recreational activities were banned, educational services were delivered online and most individuals were forced to work from home. As a result, both children with disabilities and those who were healthy were forced to stay at home with their parents for longer periods than either were accustomed to. Moreover, the rehabilitation of chronic conditions, such as cerebral palsy or neuromuscular pathologies, was mostly suspended, as in other countries in Europe [3]. Approaches such as telerehabilitation and home programs were differently activated in several areas [4,5,6], with remote clinical monitoring and psychological support ensured if needed.

Nonetheless, the burden of daily care of children with a disability that, regardless of the etiology, results in limited autonomy in daily activities and in varying levels of psychomotor delay, was left to the parents, who were also involved in schoolwork to support their children’s education [7]. This drastic change in lifestyle was probably particularly hard on adolescents with a disability; their relationships with peers, teachers and therapists, all of whom are fundamental to psychological well-being, were abruptly cut off [8,9]. Thus, this situation exacerbated the isolation of adolescents with disability and potentially threatened their mental health [10].

During this COVID-19 emergency, different authors have drawn attention to disabilities, claiming the need for inclusivity [11], warning about the risk of future increased burden of care due to reduced functional outcomes [3] and pandemic-related mental health risks [10], and have suggested an urgent rethinking about the current and future management of children with disability [12,13]. Psychological distress related to COVID-19 has been studied in the Italian population [14,15], and the implications of this pandemic for the mental health of young people has been outlined [16,17]. Additionally, the traumatic effects on families of pandemics and of the associated disease-containment measures have been confirmed globally [18,19], highlighting the need to focus on the most vulnerable families [20]. However, the perspectives of families with a child with a disability are lacking.

The aim of this study was to describe the reactions of adolescents with a disability and of parents of children with a disability to the lockdown experience, as well as their concerns and/or expectations about health care.

## 2. Materials and Methods

This was an observational cross-sectional study. Two online structured surveys were created using the free Google Forms service. These surveys were addressed to parents of children with a disability aged 0 to over 18 years and to adolescents with a disability (over age 13) to investigate their emotional reactions to the COVID-19 emergency and their concerns and expectations about health care. The links to the online surveys were sent directly to the patients and to families followed by the Children Rehabilitation Unit for Severe Developmental Disabilities of the Azienda Unità Sanitaria Locale—IRCCS of Reggio Emilia (Italy). This Unit mainly deals with children and adolescents with cerebral palsy and with neurometabolic/neuromuscular syndromes (respectively 1495 and 384 patients who accessed the Unit in the last year). The online surveys were also disseminated through professional or family associations. Data collection took place from 15 April to 15 May 2020. To comply with Italian law, which prevents the collection of data relating to the health of a minor without the authorization of their parents, the links were sent to the parents, who subsequently chose whether to involve their adolescent child in answering the questionnaire.

Both surveys were strictly anonymous at the source. Thus, based on the Italian law, the approval of the Local Ethics Committee was unnecessary. Individuals were informed that their answers would be confidential and managed in accordance with European Regulation n.679/2016 (General Data Protection Regulation) and current Italian legislation on privacy. They were then invited to express their informed consent to participate in the survey by answering the question, “Do you want to participate in this survey?” If the individuals answered “Yes,” they were allowed access to the survey. Most multiple-choice questions were mandatory to proceed, but to save and submit the answers, the participants had to select the final “Enter” button; otherwise, their answers were deleted.

The parent survey was composed of 48 multiple-choice questions and one final optional open-ended question (Supplementary Digital Material: Supplementary Table S1 online content only). The adolescent survey was composed of 32 multiple choice questions and one final optional open-ended question (Supplementary Digital Material: Supplementary Table S2 online content only). In both cases, the optional question was, “If you want, you can write any additional personal comments.”

Both surveys included questions related to demographic information (age and sex), the situation at home (number of cohabitants and amount of room in the house), consequences of the lockdown on parents’ work and the child’s/adolescent’s type of disability. It must be noted that, since it is likely that only individuals with relatively preserved cognitive function could participate in the study, the “Cognitive disability” category was an option only in the parent survey. Further questions investigated participants’ direct or indirect contact with individuals with COVID-19 and their feelings and emotions perceived during the COVID-19 outbreak. We also assessed the presence of anxiety-related symptoms based on the Generalized Anxiety Disorder 7-item (GAD-7) scale [21]. Since our objective was not to investigate the prevalence of anxiety disorders in the examined population, we preferred to contextualize the questions about emotional reactions to establish a more empathic relationship with parents and adolescents with disability and to help them express their feelings. Moreover, we assessed the participants’ main concerns related to the interruption of school, rehabilitation and free-time activities and their expectations about the future. We explored which measures they considered helpful in supporting them during and after the emergency, and whether they thought the emergency situation might bring about any positive effect.

### Statistical Analysis

We aimed to collect data from a convenience sample composed of the largest number of adolescents and parents possible who spontaneously accepted our invitation to participate. The collected data were summarized through descriptive statistics.

For each question concerning anxiety symptoms, we assigned scores of 0, 1, 2 and 3 to the response categories “Never,” “Some days,” “More than half of the days” and “Nearly every day,” respectively. We then calculated the median value of expressed emotions for each participant to represent individual anxiety symptoms. We computed a Spearman’s rank correlation test to investigate the correlation between demographic characteristics, financial and work-related problems, telerehabilitation involvement, telemonitoring by the rehabilitation team and symptoms of anxiety in each group (parents and adolescents). We computed a Mann–Whitney’s U test to compare the answers of mothers and fathers. The same test was applied to analyze the concerns of parents related to the type of disability. Statistical analyses were performed using SPSS version 26.0 statistical software (IBM).

## 3. Results

### 3.1. Demographic Information

A total of 53 adolescents and 239 parents completed the surveys. Most parents were between ages 35 and 55 (79.5%); 84.9% were female. Referring to their children, parents reported mostly multiple types of disabilities, with a high prevalence of motor disability (81%), followed by cognitive disability (48%), visual disability (13.6%), hearing disability (4.2%) and autism/autism-like (6.6%). Adolescents’ ages were almost equally distributed between age 13 years and over 18; 45.3% were female. The majority of adolescents declared multiple disabilities, mostly motor difficulties (83%), followed by general difficulties in doing things as compared to their peers (39.6%) and difficulties with schoolwork (18.9%). Three adolescents (5.7%) reported no difficulties at all. Parents’ and adolescents’ demographic information is summarized in Table 1 and Table 2, respectively.

### 3.2. Effects of the Lockdown

Both groups were asked about the positive (Table 3) and negative (Table 4) effects of lockdown. While most parents (53.6%) denied any positive effects, 92.5% of adolescents expressed positive consequences, and 64.2% of them were pleased to spend more time with their families. Conversely, only 27.2% of parents chose this option as a positive aspect.

As fallouts, 47.3% of parents were worried about their child’s disability and 40.6% complained of the excessive burden of taking care of the family. Among adolescents, the majority (73.6%) suffered from being prohibited from meeting friends.

### 3.3. Socioeconomic Aspects

Regarding economic aspects, 90 parents (37.7%) declared they were not forced to stop working because of the lockdown, while 85 (35.6%) were forced to and 64 (26.8%) changed to remote working. Among parents, 130 (54.4%) were employed, 61 (25.5%) were housewives and 32 (13.4%) were self-employed. Almost half of the parents declared that lockdown had no (13.8%) or little (29.3%) negative impact on their work. For 31.4%, lockdown had quite a negative effect, while for 13.8% the impact was considerable and for 7.1% it was extremely negative. However, only 45 parents (18.8%) reported financial difficulties since the beginning of the lockdown. Among adolescents, 29 (54.7%) declared they were aware of their family’s financial difficulties.

### 3.4. Frequency of COVID-19

Fortunately, only three (1.3%) parents and none of the adolescents with disability contracted COVID-19, while 77.2% of parents and 51% of adolescents knew at least one person who had been infected.

### 3.5. Emotional Reactions

Parents were worried about contracting COVID-19: 53.6% were quite concerned, 17.2% very concerned and 5.9% extremely worried. Conversely, 46% thought relatives getting sick was unlikely and 40.6% considered it quite likely.

Most adolescents were not (34%) or just a little (28.3%) concerned about contracting COVID-19. Conversely, 39.6% were quite concerned that a relative would get sick; this concern was considerable for 26.4% and extreme for 15.1%.

The feelings evoked by COVID-19 are represented in Table 5: in both groups “concern” prevailed, followed by “anxiety” among parents and “sadness” among adolescents.

Regarding the feelings parents experienced in the month prior to completing the survey, “concern about supporting their children in their schoolwork” was reported by 38.5% “Nearly every day” and by 25.1% “More than half of the days.” The second most frequent feeling was “nervous and/or anxious,” experienced by 20.9% “Nearly every day.” Moreover, the following feelings were perceived by some parents “More than half of the days”: difficulties relating to their relationship with spouse/partner (18.8%) or family conflicts (17.1%), fear that something terrible might happen (16.7%), being agitated and unable to be still (15.1%), oppressed either by a sense of boredom or emptiness (14.6%) or by a sense of loss or mourning (8%).

In contrast, the adolescents replied they had negative feelings predominantly “Never” or just “Some days” in the prior month, with the exception of feelings of anxiety and concern about how long the emergency would last, reported as occurring “Nearly every day” in 20.8% and 30.2%, respectively.

Examining more closely the parents’ attitudes towards their children’s future, we found a high level of concern, particularly regarding their children with a disability. Parents who also had normally developing children admitted they were worried more for their child/children with a disability than for the others in 87.8% of cases. In contrast, adolescents with a disability reported mostly low concern about their future.

Both parents and adolescents expressed concern about uncertainty regarding when the emergency would end (60.3% and 73.6%, respectively). Parents were mostly worried about reducing or stopping rehabilitative interventions for their children (63.2%), while only a few adolescents were concerned about having to stop physiotherapy (18.9%). Adolescents were predominantly concerned about being unable to see their friends (67.9%), unable to do their free-time activities (49.1%), the risk that a relative or friend might get sick (43.4%) or school being suspended (41.5%).

### 3.6. Activities during Lockdown

In spite of the lockdown, different activities were proposed to the families. As shown in Table 6, both parents and adolescents declared that schoolwork was the most frequently proposed activity (58.2% and 52.8%, respectively). They also received phone/videocalls from professionals of the rehabilitation centers (43.1% of parents and 32.1% of adolescents), but only a few took part in telerehabilitation (23.1% of parents and 32.1% of adolescents). In terms of associations and recreational/educational centers, 31.3% of parents and 22.6% of adolescents responded that these centers also began to organize follow-up phone calls and activities at home.

### 3.7. Correlation Analysis

Computing multiple Spearman’s correlation on parents’ responses, we found that the level of anxiety symptoms correlated strongly with financial problems (r_s_ 0.258, *p* = 0.000), with a negative impact of the lockdown on work (r_s_ 0.200, *p* = 0.002), with concern about contracting COVID-19 (r_s_ 0.234, *p* = 0.000) and concern about the child/children’s future (r_s_ 0.318, *p* = 0.000), particularly regarding a child with a disability (r_s_ 0.241, *p* = 0.000). However, no correlations were found with parents’ age, with the suspension of rehabilitative activities or with knowing someone affected by COVID-19.

Concern about the risk of contracting COVID-19 or about relatives contracting it showed no correlation with any of the following: knowing someone affected, work or financial problems caused by the emergency or the situation at home. We found a slight reverse correlation between parents’ age and the fear of contracting COVID-19 (r_s_ 0.594, *p* = 0.035).

An association was found between financial problems and the negative impact of lockdown on work (r_s_ 0.191, *p* = 0.003), the suspension of work because of COVID-19 (r_s_ 0.156, *p* = 0.016), the number of family members (r_s_ 0.147, *p* = 0.023) and the number of children (r_s_ 0.172, *p* = 0.008), respectively. A significant reverse correlation was found between anxiety symptoms and the perception of having enough room at home (r_s_ −0.299, *p =* 0.000). We found no significant differences between mothers and fathers with regards to work difficulties, anxiety symptoms and concerns related to their children, by means of the Mann–Whitney U test.

Parents of children with a cognitive disability expressed significantly more difficulties in managing their children’s daily needs (Z 3.779, *p* = 0.001) and greater concern about their children’s future (Z 2.255, *p* = 0.024). According to the Mann–Whitney U test, a similar effect was not detected in parents of children with motor impairment.

Evaluating multiple correlations in the adolescents’ answers by means of the Spearman test, we found a significant correlation between age and perception of financial problems (r_s_ 0.442, *p* = 0.001) and concern about the future (r_s_ 0.367, *p* = 0.007). The level of anxiety symptoms correlated strongly with the perception of financial problems (r_s_ 0.402, *p* = 0.003) and concern about the future (r_s_ 0.512, *p* = 0.000) and, at a lower but still significant level, with age (r_s_ 0.271, *p* = 0.050). Concern about the future correlated with knowing someone affected by COVID-19 (r_s 0_.437, *p* = 0.001) and the fear of personally contracting (r_s_ 0.542, *p* = 0.000) or of a relative contracting COVID-19 (r_s_ 0.558, *p* = 0.000).

### 3.8. Adolescents’ and Parents’ Suggestions

When asked about which aids they would have recommended during the lockdown imposed by the COVID-19 emergency, 20–30% of parents said more phone contact with professionals, psychological support, support for schoolwork and support from associations (Table 7). A minimal proportion of adolescents (13–17%) expected the same aids, while 32.1% answered “Nothing.” Among both groups of responders, there was a significant proportion that selected “Other options” (24.3% among parents and 28.3% among adolescents), meaning that some uncoded needs unfortunately escaped the survey.

Finally, we enquired about expectations after the COVID-19 emergency (Table 8). Both parents and adolescents appeared aware of possible difficulties and delays in resuming rehabilitative activities. The perceived need for a health checkup was greater among parents (49.4%) than among adolescents (30.2%). The need for psychological support was conversely lower among parents (20.1%) than among adolescents (30.2%).

## 4. Discussion

The emotional impact of the lockdown associated with the first wave of the COVID-19 pandemic appears to have been different on parents and on adolescents with a disability. Parents were worried that their children’s disabilities would worsen because of the suspension of physiotherapy, while adolescents were mainly concerned about being unable to see their friends and do their free-time activities.

Most adolescents (63%) declared they had no problems related to their disability during lockdown; a few even declared they had no disability. This may be attributable to different factors: immature awareness of their inabilities, intrinsic advantage of being in the family setting, assistance from family members in performing daily activities and/or the reduced number of challenging situations at home. Additionally, the lockdown forced all adolescents to use social media even more to keep in touch. Considering that most adolescents who participated in this study had motor disabilities, their physical limitations (e.g., going for a walk or playing sports with peers) became irrelevant during that time. This may have allowed adolescents with a motor disability to level the playing field. With due caution, this possible interpretation partly reverses emerging trends described in the literature, which emphasize psychological distress due to quarantine in pediatric populations with disabilities [16,22].

This study confirms the difficulties parents perceived in helping their children with online schoolwork, verified by a previous study [19], certainly amplified by the fact that parents cannot replace special education teachers. This issue has been addressed in the current second phase of the pandemic: the Italian Ministry of Education has required schools to guarantee the support of special education teachers for students with a disability. Parents also complained about the burden of full-time care for their children, particularly for those with cognitive disability, and about the increase in family conflicts due to forced coexistence; only a few parents appreciated the opportunity to spend more time with their family members. In contrast, our data showed that most adolescents with a disability appreciated the increased time spent with their parents and appreciated having more time to do their favorite at-home activities. Most likely, slowing down the pace of daily life enhanced the resources of adolescents with disabilities.

Although only 18.8% of parents declared they had financial problems, 42.3% reported a work-related negative impact of the lockdown. Remarkably, anxiety symptoms in parents were strictly related to perceived financial problems [19] and with the concern about contracting COVID-19. This concern did not correlate with age, with knowing someone affected or, conversely to previous data [14,15], with female sex. Furthermore, no correlation was found between anxiety and concern about rehabilitation activity suspension, in stark contrast with the findings of another Italian cross-sectional study, which found this association in a population of 84 caregivers of children with a wide range of neurodevelopmental disabilities [23]. Nevertheless, the parents in our study declared they were worried about this, in line with many professionals [3,12,16]. However, adolescents with a disability, like any other adolescent, were much more worried about the interruption of their recreational and social activities [8]. This might suggest that rehabilitative approaches should be rethought to make them more appealing and effective in engaging patients, thereby increasing their motivation and participation in therapy.

Among adolescents, older adolescents were more anxious, and anxiety was correlated with perceptions of financial problems and with concern about the future. This confirms the findings of another Italian study, where the financial hardship experienced by families during lockdown was associated with the psychological distress of children aged 6 to 18 years old [22].

The feeling most frequently expressed by both groups was “concern,” followed by “anxiety” by parents and “sadness” by adolescents. This is in line with parents’ sense of responsibility about the future, while adolescents are more focused on the present, which is penalized by isolation. Nonetheless, older adolescents expressed concern about the future too.

The correlations between financial problems and larger families and between feelings such as anger, a sense of emptiness, fear that something terrible may happen, stress due to limited living space and smaller dwellings are evident. However, that parents’ age correlated negatively with a negative impact on their job was unexpected. This means that younger parents reported a worse impact of lockdown on their job, but no correlation was found between age and type of job (employed or self-employed).

Concerning rehabilitation, 64% of parents and 60.4% of adolescents reported attending physiotherapy before lockdown. As Negrini et al. [3] reported during lockdown, outpatient rehabilitation care for chronic diseases was suspended to give precedence to intensive and acute care for COVID-19 patients. Professionals strived to provide telerehabilitation to the most critical situations, or, at the very least, telephone or videocall, in line with subsequent recommendations by the Italian Society of Physical Medicine and Rehabilitation [24]. This was confirmed by participants in the survey: approximately one-third of both groups were engaged in telerehabilitation; moreover, 43.1% of parents and 32.1% of adolescents received calls by professionals of the rehabilitation centers for monitoring. Our data confirm the trend reported by Bertamino et al. [25], who reported that telerehabilitation and indirect remote monitoring modalities were used in 23.5% and 42.6% of cases, respectively, interviewing 68 parents referring to the Gaslini Hospital (Genova, Italy).

Indeed, telerehabilitation turned out to be appreciated by Italian families as a strategy to receive appropriate support; however, educational and policy investments are needed to make this approach feasible in all contexts [26]. A critical aspect of using online platforms for telerehabilitation is its compatibility with the General Data Protection Regulation (EU GDPR 2016/679). During lockdown, professionals may have overcome these limitations to keep in touch with the families. However, professionals were not exempted from their personal responsibilities. Even if a specific informed consent can be created to allow telerehabilitation, this issue should be adequately addressed to support professionals in telerehabilitation programs, which can be useful beyond the current emergency.

When asked about the aids desired during the COVID-19 emergency, 30.5% of parents suggested increasing and facilitating opportunities to see professionals through strategies other than in-person appointments. Moreover, approximately one quarter requested psychological support, help with schoolwork or more support from associations. Indeed, these data confirm the need to implement policies that can sustain families by supporting childcare and education and allowing for personalized strategies [19].

Enquiring into their expectations after lockdown, most parents and adolescents appeared aware of possible delays and difficulties in resuming rehabilitative activities. Almost half of the parents and one third of the adolescents requested the possibility of assessing their rehabilitative needs with professionals, showing both a useful and proactive attitude. Almost 30% of adolescents expressed the need for psychological support. This may be due to adolescents’ tendency to focus on their emotional experiences; this data point is supported by a recent comprehensive literature review, which showed that the prevalence of mood disturbances in young people during the COVID19 pandemic ranged from 18.9% to 37.4% for anxiety symptoms and from 22.6% to 43.7% for depression symptoms [17].

Regarding the final open-ended question, few adolescents answered it, and those few reported feelings of loneliness and fear of contracting COVID-19. Among the 38 parents who answered that question, the most recurrent negative word was “loneliness.” Some parents complained about the lack of support provided by rehabilitation professionals; others, instead, made the most of assistance via telerehabilitation or remote monitoring. Based on our data, which are confirmed by the open-ended questions, and perfectly in line with what a large Chinese cross-sectional survey claimed [27], it seems that the families most penalized were those with children affected by cognitive or behavioral disability.

### Study Limitations

The results of this study should be interpreted with caution as they derive from a cross-sectional design, which makes causal inferencing unadvised. Additionally, due to the limited time available to conduct the investigation, we could not pilot test the questionnaires, nor were we able to examine their psychometric properties before using them. Thus, the results obtained should be interpreted with caution. Moreover, the convenience samples of parents and adolescents were largely recruited through invitations sent to the families usually followed by the Unit that promoted this study, which mainly deals with children and adolescents with cerebral palsy and with neurometabolic/neuromuscular syndromes. This explains the prevalence of motor disability in the selected sample, which cannot be considered representative of the whole spectrum of disabilities in the developmental age.

Moreover, the surveys were available online and only in Italian. Thus, individuals that do not habitually use the Internet and those with difficulties in reading and writing Italian have probably been excluded from the survey. Furthermore, since it is likely that these questionnaires could only be completed by individuals with relatively preserved cognitive function, the views of children and adolescents with cognitive disabilities in this study were probably represented only through the answers given by their parents.

However, this sampling strategy was the only one feasible during lockdown and, even though the sample size cannot be considered representative of the considerable, highly heterogeneous target population, this study had relatively high acceptance in our community. Surely, given the variability of the ages and types of disabilities represented, future studies with larger and/or more homogeneous samples could explore this issue, with a greater likelihood of furnishing more specific indications.

## 5. Conclusions

The first wave of COVID-19 outbreak provoked devastating consequences in terms of morbidity and mortality. Furthermore, the associated lockdown had a dramatic socioeconomic impact [28]. From our small sample, while families with children with a disability appeared to be only marginally affected by COVID-19, the higher level of anxiety among parents and adolescents was associated with the fear of contracting COVID-19. The principal determinant factors were financial problems and concern about the future, particularly in larger families. These concerns are probably shared by parents of healthy children.

The lockdown in early spring 2020 deprived young people with disability of their usual stimulating social environment. This appeared particularly troublesome for those with cognitive and behavioral disabilities, despite efforts by health and school professionals to propose home activities. However, adolescents with motor disability, thanks to online platforms and assistive technology, felt the limitation on their social relationships less. Thus, it seems crucial to implement opportunities to invest massively in assistive technology devices and facilities, such as rehabilitation platforms and exergaming with remote audiovisual interactions. This approach may even result in increasing adolescents’ engagement in rehabilitative activities and empowerment. Based on our data, an effort to improve telemonitoring and telerehabilitation was made, which supported 60–70% of participants. Nonetheless, the offer needs to be enriched, differentiated and, more importantly, evenly distributed among rehabilitation services.

## Figures and Tables

**Table 1:** Parents’ demographic information (*n* = 239).

Demographics		Number (%)
Sex	Male	36 (15.1)
	Female	203 (84.9)
Age (years)	Under 35	21 (8.8)
	35–55	190 (79.5)
	Over 55	28 (11.7)
Family members	Two	15 (6.3)
	Three	70 (29.3)
	Four	101 (42.3)
	Five	41 (17.2)
	Six or more	12 (5)
Age of child/children with disability (years)	0–5	57 (22.1)
	6–10	54 (20.9)
	11–13	31 (12)
	14–22	84 (32.6)
	Over 22	32 (12.4)
Type of disability	Motor	209 (81)
	Cognitive	124 (48)
	Visual	35 (13.6)
	Hearing	11 (4.2)
	Autism	17 (6.6)
Activities before COVID-19	School	186 (77.8)
	Sports or free-time activities	127 (53.1)
	Day community services	40 (16.7)
	Physiotherapy	153 (64)
Rooms at home	1	3 (1.3)
	2	21 (8.8)
	3	77 (32.2)
	4 or more	138 (57.7)
Enough room at home	None at all	11 (4.6)
	Little	26 (10.9)
	Quite a lot	111 (46.4)
	A lot	91 (38.1)

**Table 2:** Demographic information of the adolescents with disability (*n* = 53).

Demographics		Number (%)
Sex	Male	29 (54.7)
	Female	24 (45.3)
Age (years)	13–15	17 (32)
	16–18	18 (34)
	Over 18	18 (34)
Siblings	One	23 (43.4)
	Two	8 (15.1)
	Three	5 (9.4)
	Four	2 (3.8)
	Five or more	0 (0)
	None	15 (28.3)
Type of disability	Motor	44 (83)
	Visual	2 (3.8)
	Hearing	0 (0)
	Schoolwork	10 (18.9)
	Do things as peers do	21 (39.6)
	Play with peers	4 (7.5)
	Other difficulties	6 (11.3)
	None	3 (5.7)
Activities before COVID-19	School	40 (75.5)
	Sports or free-time activities	38 (71.7)
	Day community services	4 (7.5)
	Physiotherapy	32 (60.4)
Enough room at home	Yes	49 (92.5)
	No	4 (7.5)

**Table 3:** Positive aspects of lockdown.

Responders	Aspect	Number (%)
Parents	More time spent with family members	65 (27.2)
	More time for activities of daily living	19 (7.9)
	More time to take care of child/children	27 (11.3)
	Better quality of family relationships	31 (13)
	Greater effort to understand child/children’s needs	37 (15.5)
	Other reasons	14 (5.9)
	None	128 (53.6)
Adolescents with disability	More time spent with family members	34 (64.2)
	More time for favorite at-home activities	27 (50.9)
	More time to chat with friends (tablet)	12 (22.6)
	More time to talk with friends (smartphone)	11 (20.8)
	More time to play games	12 (22.6)
	Other reasons	10 (18.9)
	None	4 (7.5)

**Table 4:** Fallout of the lockdown.

Responders	Fallout	Number (%)
Parents	Excessive burden of taking care of family members at home all day	97 (40.6)
	Increased stress caused by forced coexistence	35 (14.6)
	Worrying about child/children’s disability	113 (47.3)
	Other reasons	49 (20.5)
	None	33 (13.8)
Adolescents with disability	Attending classes online	18 (34)
	Lockdown at home, all together, all the time	26 (49.1)
	Being forbidden to meet friends	39 (73.6)
	Being forbidden to do sports or favorite free-time activities	28 (52.8)
	Being prohibited to go out	30 (56.6)
	Other reasons	5 (9.4)
	None	0 (0)

**Table 5:** Feelings evoked in parents and adolescents when thinking about COVID-19.

Responders	Fear N (%)	Anxiety N (%)	Concern N (%)	Sadness N (%)	Anger N (%)	I Do Not Know N (%)
Parents	71 (29.7)	114 (47.7)	194 (81.2)	68 (28.5)	39 (16.3)	12 (5)
Adolescents	15 (28.3)	20 (37.7)	34 (64.2)	23 (43.3)	16 (30.2)	3 (5.7)

**Table 6:** Activities proposed during lockdown.

Activities	Parents N (%)	Adolescents N (%)
Phone/videocalls from physician or physiotherapist	103 (43.1)	17 (32.1)
Telerehabilitation via videocall or sent exercise schedules	54 (22.6)	10 (18.9)
Telerehabilitation via online activities or apps	12 (5)	7 (13.2)
Phone/videocalls from associations or educational/recreational centers	46 (19.2)	4 (7.5)
Educational/recreational activities organized by associations or centers	29 (12.1)	8 (15.1)
Schoolwork required by the teachers	139 (58.2)	28 (52.8)
Nothing	25 (10.5)	10 (18.9)

**Table 7:** Aids suggested during the lockdown.

Suggested Aids	Parents N (%)	Adolescents N (%)
Increased opportunity to contact professionals	73 (30.5)	7 (13.2)
Psychological support	64 (26.8)	7 (13.2)
More support for schoolwork	71 (29.7)	9 (17)
More support from communities or associations	50 (20.9)	7 (13.2)
Other options	58 (24.3)	15 (28.3)
Nothing	29 (12.1)	17 (32.1)

**Table 8:** Measures expected after lockdown.

Expected Measures	Parents N (%)	Adolescents N (%)
Assessing their rehabilitative needs with professionals	118 (49.4)	16 (30.2)
Opportunity to express and share concerns and fears felt in this period (psychological support)	48 (20.1)	16 (30.2)
Awareness of difficulties in reorganizing rehabilitative activities because of safety measures linked to COVID-19	151 (63.2)	18 (34)
Awareness of possible delays in resuming activities	164 (68.6)	37 (69.8)
I have not thought about it	5 (2.1)	9 (17)

## Data Availability

The dataset generated and analyzed is available upon reasonable request from the corresponding author.

## Supplementary Materials

The following are available online at https://www.mdpi.com/1660-4601/18/4/1687/s1, Table S1: Parent survey, Table S2: Adolescent survey.
