# Supplementary material for: How Did Italian Adolescents with Disability and Parents Deal with the COVID-19 Emergency?
Source: Int J Environ Res Public Health. 2021 Feb 10;18(4):1687. doi: 10.3390/ijerph18041687 (PMC7916390; doi:10.3390/ijerph18041687)
Supplement: Supplementary file 1 [file ijerph-18-01687-s001.pdf]

Supplementary Table S1. Parent survey

1. Age	< 35 y.o.
	35-55 y.o.
	> 55 y.o.
2. Sex	Male
	Female
3. How many people are there in your family?	Two/ Three/ Four / Five
	Six
4. How many children do you have?	Two/ Three/ Four
	Five or more
5. How many children with disability do you have?	One / Two/ Three
	>Three
6. Sex of the first disabled child	Male
	Female
7. Age of the first disabled child	< 6 y.o.
	6-10 y.o.
	11-13 y.o.
	14-22 .o.
	> 22 y.o.
8. Type of disability of the first disabled child *(multiple answers allowed)*	Motor disability
	Cognitive disability
	Motor and cognitive disability
	Visual disability
	Hearing disability
	Autism spectrum disorder
9. Sex of the second disabled child *(optional)*	Male
	Female
10. Age of the second disabled child *(optional)*	< 6 y.o.
	6-10 y.o.
	11-13 y.o.
	14-23 .o.
	> 22 y.o.
11. Type of disability of the second disabled child *(optional; multiple answers allowed)*	Motor disability
	Cognitive disability
	Motor and cognitive disability
	Visual disability
	Hearing disability
	Autism spectrum disorder
12. Sex of the third disabled child *(optional)*	Male
	Female

13. Age of the third disabled child *(optional)*	< 6 y.o.
	6-10 y.o.
	11-13 y.o.
	14-24 .o.
	> 22 y.o.
14. Type of disability of the third disabled child *(optional; multiple answers allowed)*	Motor disability
	Cognitive disability
	Motor and cognitive disability
	Visual disability
	Hearing disability
	Autism spectrum disorder
15. Which activities was your disabled child used to attend? *(multiple answers allowed)*	School
	Sports or other recreational activities
	Day-time community services
	Physiotherapy
16. Has the stopping of these activities induced any positive effect on daily life management? *(multiple answers allowed)*	Yes, more time spending with relatives
	Yes, more time to carry out daily life activities
	Yes, more time to take care of my child/children
	Yes, higher quality of family relationships
	Yes, more participation in understanding my disabled child/children' s needs
	Yes, other reasons
	None
17. Has the stopping of these activities induced any negative effect on daily life management? *(multiple answers allowed)*	Yes, excessive burden of taking care of relatives staying at home all day
	Yes, much stress caused by forced coexistence
	Yes, worrying about my child/children's disability
	Yes, other reasons
	None
18. How many rooms are there in your home, apart from the bathroom ?	One / Two / Three
	Four or more
19. Considering the lockdown, do you think that spaces in your home are enough for an acceptable coexistence?	Not at all
	Little
	Quite
	A lot
20. What kind of job do you do?	Freelance
	On a salary
	Outside contractor
	Unemployed
	Housewife

21. Have you stopped working because of COVID-19 emergency?	Yes
	No
	Converted into smart working
22. Does COVID-19 affect negatively your job?	Not at all
	Little
	Quite
	A lot
	Extremely
23. Have you experienced any financial difficulty since the beginning of COVID-19 emergency?	Yes
	No
24. Do you know someone who contracted COVID-19? *(multiple answers allowed)*	Me
	One or more relatives
	On or more colleagues
	One or more acquaintances
	No
25. How much worried are you of contracting COVID-19?	Not at all
	Little
	Quite
	A lot
	Extremely
26. In your opinion, how likely is it that one of your relatives can contract COVID-19?	Not at all
	Little
	Quite
	A lot
	Extremely
27. How much do you think your relatives are worried of contracting COVID-19?	Not at all
	Little
	Quite
	A lot
	Extremely
28. Which of the following emotions do you feel when thinking about COVID-19? *(multiple answers allowed)*	Fear
	Anxiety
	Concern
	Sadness
	Anger
29. How frequently have you felt more nervous and/or anxious thinking to your child's disability, in the past month?	Never
	Some days
	More than half the days
	Nearly every day

30. How frequently have you worried about supporting your child/children in schoolwork, in the past month?	Never
	Some days
	More than half the days
	Nearly every day
31. How frequently have you worried excessively about financial or job difficulties, in the past month?	Never
	Some days
	More than half the days
	Nearly every day
32. How frequently have you had troubles in managing your disabled child/children day life needs, in the past month?	Never
	Some days
	More than half the days
	Nearly every day
33. How frequently have you felt stressed because of restricted spaces and seclusion in your home?	Never
	Some days
	More than half the days
	Nearly every day
34. How frequently have you got easily irritated in your couple relationship, in the past month?	Never
	Some days
	More than half the days
	Nearly every day
35. How frequently have you worried about managing family conflicts, in the past month?	Never
	Some days
	More than half the days
	Nearly every day
36. How frequently have you experienced fear that something terrible could happen, in the past month?	Never
	Some days
	More than half the days
	Nearly every day
37. How frequently have you felt oppressed by a sense of boredom or emptiness, in the past month?	Never
	Some days
	More than half the days
	Nearly every day
38. How frequently have you felt oppressed by a sense of loss or mourning, in the past month?	Never
	Some days
	More than half the days
	Nearly every day
39. How frequently have you had difficulties in relaxing, in the past month?	Never
	Some days
	More than half the days
	Nearly every day

40. How frequently have you been agitated and unable to stay still, in the past month?	Never
	Some days
	More than half the days
	Nearly every day
41. How much worried are you about your child/children's future?	Not at all
	Little
	Quite
	A lot
	Extremely
42. How much worried are you about your disabled child/children's future?	Not at all
	Little
	Quite
	A lot
	Extremely
43. If you have more than one child, do you perceive any difference in your worrying about your disabled child compared to the other/s? *(optional; multiple choice accepted)*	Not at all
	Little
	Quite
	A lot
	Extremely
44. What do you mostly worry about? *(multiple choice accepted)*	I don't know when this emergency situation will end
	Reduced follow-up by Health Services for my disabled child/children
	Worsened sanitary assistance provided to my child
	The consequences of the need of new procedures and new devices for safety and infection prevention
	The chance of reducing supports for school, educational or recreational activities for my disabled child/children
	Nothing
45. Which steps have been taken by health professionals during this period? *(multiple choice accepted)*	Phone or videocalls by the physician or the physiotherapist to check the situation and collect any need
	Telerehabilitation by videocalls or sending exercise schedules
	Telerehabilitation by online activities or apps
	Phone or videocalls by professionals or volunteers from associations or educational/recreational communities
	Educational/recreational activities remotely offered by volunteers or professionals from associations or communities
	Schoolwork requested by the teachers
	Nothing

46. Which aids do you think could help you during COVID-19 pandemic?<br>*(multiple choice accepted)*	Increased opportunity to reach my child/children's physician or physiotherapist, by phone or videocalls
	Opportunity to have phone or videocalls for psychological support
	More support to my disabled child/children for schoolwork
	More support by volunteers or professionals from communities or associations usually attended by my child/children
	Other options
	Nothing
47. How do you think will the rehabilitative, educational and recreational activities be reorganized after COVID-19 emergency?<br>*(multiple choice accepted)*	It would be useful to check the health situation of my child/children, with physician or physiotherapist, for any new needs
	It would be useful to have the opportunity to express and share concerns and fears felt in this period
	I realize that there may be difficulties in reorganizing the rehabilitation connected activities, because of cautions and safety measures linked to COVID-19
	I realize that it will take some time to restart activities
	I haven't thought about it
48. Do you think there could be any positive effect resulting from the COVID-19 emergency?<br>*(multiple choice accepted)*	Improvement of communication with physicians, physiotherapists, other professionals
	Slowing down with job-home-recreational activities
	No improvement
	Other options
49. If you want you can write any additional personal comments	………………………………………………………………

Supplementary Table S2. Adolescent survey

1. Age	13-15 y.o.
	16-18 y.o.
	> 18 y.o.
2. Sex	Male
	Female
3. How many sisters/brothers?	One/ Two/ Three/ Four
	Five or more
	None
4. Which difficulties connected to your disability?	Difficulties in motor activities as walking, standing (motor disability)
	Difficulties in seeing (visual disability)
	Difficulties in hearing (hearing disability)
	Difficulties in accomplishing activities as peers
	Difficulties in playing with my schoolmates or with people that I'm not familiar with
	Difficulties in schoolwork
	Other difficulties
	None
5. Which activities were you used to attend before the the lockdown due to COVID-19 emergency? *(multiple answers allowed)*	School
	Sports or other recreational activities
	Day-time community services
	Physiotherapy
6. Which of these activities do you miss the most? *(multiple answers allowed)*	School
	Sports or other recreational activities
	Day-time community services
	Physiotherapy
	Other
	None
7. Has the stopping of these activities induced any positive effect? *(multiple answers allowed)*	Yes, more time spending with relatives
	Yes, more time to carry out my preferred home activities
	Yes, more time to chat with friends by tablet
	Yes, more time to talk with friends by smartphone
	Yes, more time to play games
	Yes, other reasons
	None
8. Has the stopping of these activities induced any negative effect? *(multiple answers allowed)*	Yes, attending school lessons online
	Yes, lockdown at home, all together, all the time
	Yes, being forbidden to meet friends

	Yes, being forbidden to do sports or my preferred free-time activity
	Yes, being prohibited to get out
	Yes, other reasons
	None
9. Do you have a place to stay by yourself, without excessive stress?	Yes
	No
10. Are you pleased enough of how you are spending your time during this lockdown?	Not at all
	Little
	Quite
	A lot
11. Have you heard about financial difficulty since the beginning of COVID-19 emergency?	Yes
	No
12. Do you know someone who contracted COVID-19? *(multiple answers allowed)*	Me
	One or more relatives
	One or more friends or acquaintances
	No
13. Are you worried about contracting COVID-19?	Not at all
	Little
	Quite
	A lot
	Extremely
14. Are you worried that one of your relatives may contract COVID-19?	Not at all
	Little
	Quite
	A lot
	Extremely
15. How much do you think your relatives are worried of contracting COVID-19?	Not at all
	Little
	Quite
	A lot
	Extremely
16. Which of the following emotions do you feel when thinking about COVID-19? *(multiple answers allowed)*	Fear
	Anxiety
	Concern
	Sadness
	Anger
	Never
	Some days

17. How frequently have you felt more nervous and/or anxious, in the past month?	More than half the days
	Nearly every day
18. How frequently have you worried about schoolwork, in the past month?	Never
	Some days
	More than half the days
	Nearly every day
19. Are you worried about the lasting of this emergency situation?	Never
	Some days
	More than half the days
	Nearly every day
20. Have you complained difficulties because of your disability in this period?	Never
	Some days
	More than half the days
	Nearly every day
21. Have you felt stressed because of restricted spaces and seclusion in your home?	Never
	Some days
	More than half the days
	Nearly every day
22. Have you perceived difficulties in the relationships with your relatives, in this period?	Never
	Some days
	More than half the days
	Nearly every day
23. Have you experienced fear that something terrible could happen?	Never
	Some days
	More than half the days
	Nearly every day
24. Have you felt oppressed by a sense of boredom or emptiness?	Never
	Some days
	More than half the days
	Nearly every day
25. Have you had difficulties in relaxing?	Never
	Some days
	More than half the days
	Nearly every day
26. Have you been agitated and unable to stay still?	Never
	Some days
	More than half the days
	Nearly every day
27. Are you worried about your future?	Not at all

	Little
	Quite
	A lot
	Extremely
28. What are you worried about most, in this period?	Ignoring when this situation will finish
	School stop
	Sport and free-time activities stop
	Being forbidden to meet friends
	Physiotherapy stop
	Getting sick
	Some relative or friend getting sick
	Nothing
29. Which activities have been proposed to you during this period? *(multiple choice accepted)*	Phone or videocalls by my physician or my physiotherapist to know how I am doing
	Telerehabilitation by videocalls or sending exercise schedules
	Telerehabilitation by online activities or apps
	Phone or videocalls by professionals or volunteers from associations or educational/recreational communities
	Educational/recreational activities remotely offered by volunteers or professionals from associations or communities
	Schoolwork requested by the teachers
	Nothing
30. Which aids do you think could help you during COVID-19 pandemic? *(multiple choice accepted)*	Phone or videocalls to reach my physician or physiotherapist
	Phone or videocalls for psychological support
	More support for schoolwork
	Phone or videocalls from volunteers or professionals from communities or supporting associations
	Other options
	Nothing
31. What do you think could be done after COVID-19 emergency? *(multiple choice accepted)*	It would be useful to check my health situation with my physician or physiotherapist
	It would be useful to have the opportunity to express and share concerns and fears felt in this period
	I realize that there may be difficulties in reorganizing the rehabilitation connected activities, because of cautions and safety measures linked to COVID-19
	I realize that it will take some time to restart activities
	I haven’t thought about it

32. Do you think there could be any improvements resulting from the COVID-19 emergency? *(multiple choice accepted)*	Improvement of friendships
	Improvement of family relationships
	No improvement
	Other options
33. If you want you can write any additional personal comments	……………………………………………………………….
